# Deep learning image segmentation and extraction of blueberry fruit traits associated with harvestability and yield

**DOI:** 10.1038/s41438-020-0323-3

**Published:** 2020-07-01

**Authors:** Xueping Ni, Changying Li, Huanyu Jiang, Fumiomi Takeda

**Affiliations:** 1 College of Biosystems Engineering and Food Science, Zhejiang University, Hangzhou, China; 2 College of Engineering, University of Georgia, Athens, GA USA; 3 Appalachian Fruit Research Station, USDA-ARS, Kearneysville, WV USA

**Keywords:** Plant breeding, Plant breeding

## Abstract

Fruit traits such as cluster compactness, fruit maturity, and berry number per clusters are important to blueberry breeders and producers for making informed decisions about genotype selection related to yield traits and harvestability as well as for plant management. The goal of this study was to develop a data processing pipeline to count berries, to measure maturity, and to evaluate compactness (cluster tightness) automatically using a deep learning image segmentation method for four southern highbush blueberry cultivars (‘Emerald’, ‘Farthing’, ‘Meadowlark’, and ‘Star’). An iterative annotation strategy was developed to label images that reduced the annotation time. A Mask R-CNN model was trained and tested to detect and segment individual blueberries with respect to maturity. The mean average precision for the validation and test dataset was 78.3% and 71.6% under 0.5 intersection over union (IOU) threshold, and the corresponding mask accuracy was 90.6% and 90.4%, respectively. Linear regression of the detected berry number and the ground truth showed an *R*^2^ value of 0.886 with a root mean square error (RMSE) of 1.484. Analysis of the traits collected from the four cultivars indicated that ‘Star’ had the fewest berries per clusters, ‘Farthing’ had the least mature fruit in mid-April, ‘Farthing’ had the most compact clusters, and ‘Meadowlark’ had the loosest clusters. The deep learning image segmentation technique developed in this study is efficient for detecting and segmenting blueberry fruit, for extracting traits of interests related to machine harvestability, and for monitoring blueberry fruit development.

## Introduction

Southern highbush (SHB) blueberries (a group of hybrid cultivars developed from crossing *Vaccinium corymbosum* L., *V. darrowi* Camp, and other *Vaccinium* species indigenous to southeastern United States) are adapted to areas with mild winters. SHB blueberry production in tropical and subtropical environments has become an economically important crop in Florida, North Carolina, and Georgia in the Southeastern United States as well in California, Mexico, Peru, Australia, northern Chile, and North Africa. At present, most SHB blueberries destined for the fresh market are still hand harvested, but they also are harvested mechanically during the latter part of the season when it is no longer economical to harvest by hand^1^. Mechanically harvested fruit may either go to the fresh or processed market depending on the cultivar and condition of the fruit. To reduce labor costs, blueberry growers in Florida and Georgia, in other production areas across the United States, and in major blueberry producing countries are showing an increasing interest in adopting mechanical harvesters for harvesting blueberries intended for the fresh market^2,3^. Among the many plant and fruit characteristics that blueberry breeders evaluate in a cultivar intended for mechanical harvest are plant vigor, a narrowly upright plant canopy, and a firm fruit with a dry scar^4^. In addition, fruit should be produced in a loose cluster along with concentrated ripening^5^. Compact or tight fruit clusters make it difficult to selectively harvest ripe fruit among immature fruit in a cluster either by hand or machine.

In grapes, fruit maturation among bunches (e.g., clusters) vary, but the individual berries on a bunch ripen uniformly. Table grapes are harvested as bunches in several pickings, and in wine and raisin grapes all bunches are harvested in an once-over picking operation. Many studies have used the International Organization of Vine and Wine (OIV) descriptor^6^ to classify differences in grape cluster compactness, which is nevertheless still a subjective method for dealing with all aspects of grape production. There are also many objective methods to quantify compactness. Tello et al.^7^ used a quantitative grape compactness index (CI13), which is calculated as the ratio of the berry volume over the square of rachis length. Cubero et al.^8^ defined the ratio of the square of the berry perimeter over the berry area of the grape bunch for bunch compactness and found that a smaller perimeter with the same area would be more compact. The perimeters and areas of grape bunches were extracted from 2D images. Rist et al.^9^ compared the correlation of five different factors with the OIV grape compactness descriptor, including (a) the ratio of the number of berries and grape length, (b) the ratio of total berry volume and the sum of the grape length and grape width, (c) the ratio of berry volume and grape length, (d) the difference of convex hull volume and total berry volume, and (e) the ratio of convex hull volume and the sum of grape length and grape width. They found that the order from the highest correlation to the lowest was factor (b), (c), (a), (e), and (d). All the parameters in these five factors were extracted from the 3D grape bunch point cloud using a 3D scanner. Hed et al.^10^ used the number of berries per centimeter of rachis as a grape compactness index. However, these methods did not provide high throughput and required significant manual operations.

In blueberries, there is a large variation in fruit maturation among clusters, fruit within a cluster, and among branches of the bush. This means that mature fruit must be harvested selectively after each fruit develop a blue color. Therefore, evaluating the maturity of individual blueberry fruit can provide valuable information for determining the best harvesting time. Number counting and cluster tightness are also important in the blueberry industry and can be used to estimate yield and harvesting strategy. Thus, an automated method to characterize these three traits (compactness, maturity, and count) and present the analysis would be a valuable tool for blueberry breeders as well for blueberry growers.

Compactness is an important index for quantifying the tightness of blueberry clusters. Schwartze et al.^11^ reported that small and loose clusters ripened more evenly and were easier to hand harvest than those with long and tight clusters. During mechanical harvesting, blueberries on a loose cluster in response to external vibration can oscillate about its pedicel in three ways (swing, tilt, and rotate)^1^. In contrast, blueberries in a tight cluster would not oscillate, as each fruit is pressed against two or three berries. It can be inferred that loose blueberries are more suitable for mechanical harvesting when other factors are the same^5,12^. However, there is currently no official blueberry compactness definition, and only a few related studies have appeared. Brightwell^13^, for instance, defined three levels of compactness for blueberry clusters: compact, medium compact, and loose. The compact clusters are those in which all berries touch each other and in which the first ripe berry is difficult to pick, either by hand or with a machine. The medium compact clusters are those in which most berries are touched by two or more fruits and the first ripe berry is difficult to dislodge. The loose clusters are those in which most berries may or may not be touching other berries and are easy to pick. Brightwell’s definition, however, is subjective and difficult to quantify. A solution for automatic compactness extraction is, therefore, necessary and desirable for blueberry breeding programs and increasing production and harvest efficiency.

Previous literature has shown that although the maturity of blueberry fruits is defined by their color, maturity definitions have differed. Gilbert et al.^14^ classified blueberry fruits into four different developmental stages: (A) green, (B) breaker, (C) red, and (D) blue. El Agamy et al.^15^ defined more stages according to the fruit color: (a) green-mature, (b) green + 25% pink, (c) 50% pink, (d) 75% pink, (e) pink, (f) bluish pink (pink + 50% blue), (g) 75% blue, (h) blue, and (i) blue-black, in which the maturity stage (h) is for commercial picking and stage (i) is beyond commercial handling. Kalt et al.^16^ studied the relationship between surface color and other maturity indices (size, sugar, acid, and anthocyanin contents). The results showed that sugar content was highly correlated with the surface color, indicating that the surface color can represent the berry’s maturity. Yang et al.^17^ showed the spectral differences among young fruits (green color), intermediate fruits (red color), and mature fruits (dark blue/purple). In all these studies, the fruit maturity index was assigned to individual blueberries. For this paper, the individual berry maturity property was classified and then the maturity ratio of the whole branch was calculated.

Various traditional methods have been used for berry detection and classification. One popular method has been to use the spectral properties of blueberries. Yang et al.^18^ measured spectral reflectance of blueberry leaves, mature fruits, near-mature fruits, near young mature fruits, and young fruits for different cultivars. Classification models were established, and the model achieved more than 98% accuracy for all leaves and fruits. However, the application of this method is too limited, as it only can be used for indoor research with artificial lighting and under a cover that blocks out ambient light to distinguish leaves and different maturities of blueberries. One of our goals was to segment individual blueberries and assess individual fruit maturity and cluster maturity indices when capturing RGB (red, green, and blue) images outdoor.

As a deep learning technique, convolutional neural networks (CNNs) have shown great promise for image classification, object detection, and segmentation^19^. One successful application of CNN is using semantic segmentation to classify objects on images at the pixel level^20^. Since Papandreou et al.^20^ proposed semantic image segmentation using CNN achieving a mean intersection over union (mIOU) of over 70% on PASCAL Visual Object Classes (VOC) 2012 dataset, many other models have been developed. Long et al.^21^ proposed a fully convolutional network (FCN), whose output images have the same resolution as input images while achieving an mIOU of 62.2% on PASCAL VOC dataset. FCNs were then improved in some advanced models. U-Net^22^ was developed based on an FCN with data augmentation, achieving an mIOU of over 92% on the biomedical dataset with very few training images. However, U-Net segments all objects as a union mask when Mask R-CNN can predict objects as individual masks. Mask R-CNN^23^ combines Faster R-CNN^24^ and FCN and can achieve a higher accuracy than when only using FCN. DeepLab^25^ was developed to deal with multiple scales of objects (objects with different sizes) without reducing the resolution and produced an mIOU of 79.7% on PASCAL VOC-2012. DeepLabv3^26^ and DeepLabv3+^27^ were developed by improving DeepLab and achieved an mIOU of 85.7% and 89% on PASCAL VOC-2012, respectively. FastFCN^28^ proposed a joint pyramid up-sampling that can extract high resolution feature maps three times faster without performance losses. Semantic segmentation for videos was also developed achieving an mIOU of 83.5% on a Cityscapes dataset and 82.9% on the Cambridge-driving Labeled Video Database (CamVid) dataset^29^. For this paper, we used Mask R-CNN to segment individual berries given its superior performance.

Some semantic segmentation models have been used on berry segmentation. Milella et al.^30^ used four different CNNs to segment grape bunches, leaves, wood (grapevine trunks, cordons, and canes), and vineyard grape poles from the background in the RGB images and concluded that using an VGG19^31^ network had the best performance, with an accuracy of 91.52%. Santos et al.^32^ compared Mask R-CNN^23^, YOLOv2^33^, and YOLOv3^34^ to detect and segment grape bunches in the field. However, both studies segmented the whole grape bunch but not individual berry segmentation to count berries. To detect and segment individual grapes, Zabawa et al.^35^ used an FCN^21^ to detect the edges of a single berry from color images to obtain a single grape berry. Some studies^35,36^ have been conducted on individual grape detection and segmentation using deep learning, but no study has been done on blueberries that have a significantly different growth pattern and fruit characteristics from grapes. Compared with grape detection, blueberry detection is more challenging because the fruits spread throughout the branches and the maturity of fruits in one branch can differ even in one cluster.

The main goal of this study was to apply Mask R-CNN for blueberry fruit segmentation to calculate harvestability and yield traits. Specific objectives were to:

1. Segment individual berries using Mask R-CNN with an iterative annotation method and evaluate the classification and segmentation performance.
2. Define and calculate blueberry maturity and compactness and count blueberry number per clusters.
3. Assess the accuracy of the extracted traits and delineate trait differences in four blueberry cultivars.

## Results

### Segmentation and trait extraction results

**Fig. 1:**
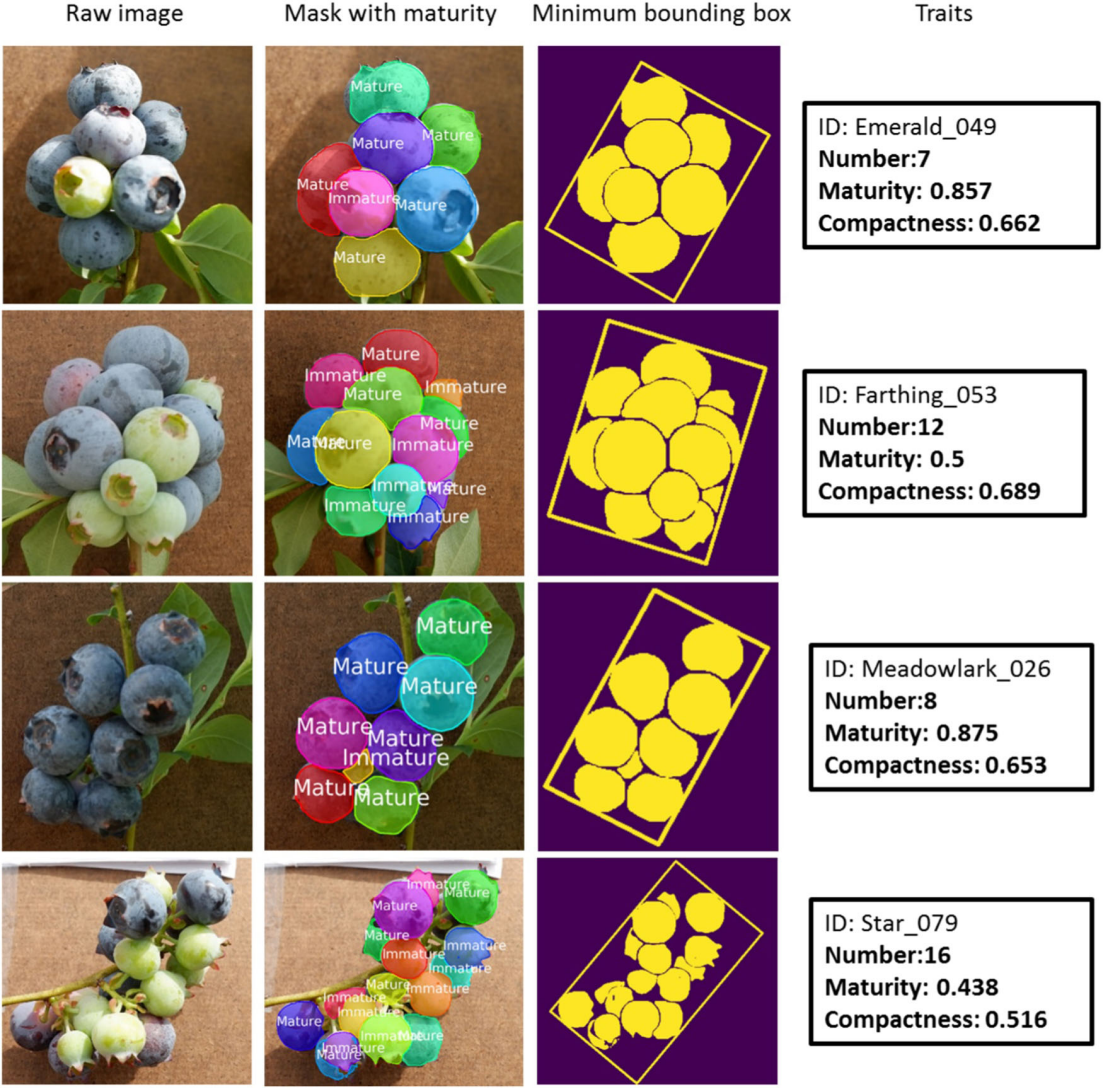
Detection examples for each cultivar with traits extraction. The black rectangle contains the ID number and the three traits (number, maturity, and compactness) within the corresponding sample

The trained berry detection model was used to segment individual berries according to maturity and to extract other relevant traits. Figure 1 shows four test images’ berry detection results. These images represent one of the samples of each cultivar. The detected blueberry mask with a minimum bounding box is also shown in the result. The traits were calculated from the detection results. Compared with the raw images, the mask accurately covered the berries, including some berries that were only partially visible behind the berries in the foreground. The numbers detected for ‘Emerald’_049 and ‘Meadowlark’_026 were identical with the ground truth in the raw images. Both numbers detected for ‘Farthing’_053 and ‘Star’_079 were one berry less than the ground truth in the raw images. The maturity of almost all berries was detected accurately except for the berry in ‘Meadowlark’_026, where a small part of the mature berry on the back was detected as immature. Regarding compactness, although there was no ground truth to assess the accuracy of the image-derived values, the differences in the compactness values of the four cultivars generally matched with our visual intuition.

### Detection and segmentation performance evaluation

**Fig. 2:**
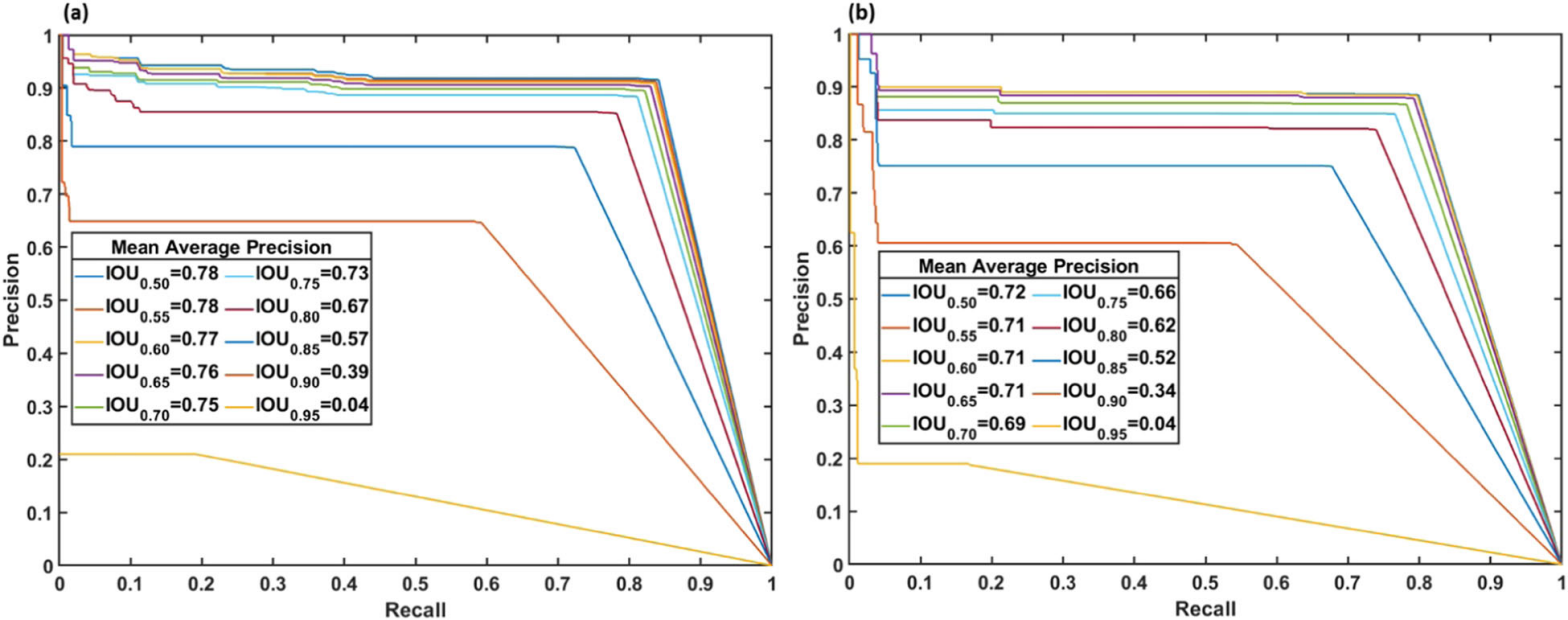
Recall–precision curve of various models under different IOU thresholds. **a** Validation and **b** test datasets.

To evaluate the performance of the detection model, the mean average precision (mAP) and mIOU were calculated and analyzed. The model of epoch 510 was selected, because it had a relatively low validation loss. Under the 0.5 IOU threshold, the mAP of the validation dataset (145 images with thousands of berries) was 0.783 and 0.716 for the test datasets (55 images with hundreds of berries). From Fig. 2, it can be seen that with an IOU threshold greater than 0.85, the mAP was lower than 0.5 in both datasets. When the IOU threshold equaled 0.95, mAP was close to zero for both the validation and test datasets because many berries could not be detected with a high IOU threshold. It is obvious that a higher IOU threshold results in higher precision but with fewer detected objects. In many cases, researchers have used the IOU threshold of 0.5 to calculate average precision (AP). Because compactness was calculated from the mask area and minimum bounding box area (determined by the mask), it was necessary to evaluate mask accuracy. The mIOU was used to indicate the berry mask accuracy further for better compactness accuracy. The mIOU of the validation dataset and test dataset were 0.906 and 0.904 with an IOU threshold of 0.5, respectively, which indicated that 90.6% and 90.4% of the berry mask areas match the ground truth for the validation and test dataset, respectively.

### Iterative annotation performance

**Fig. 3:**
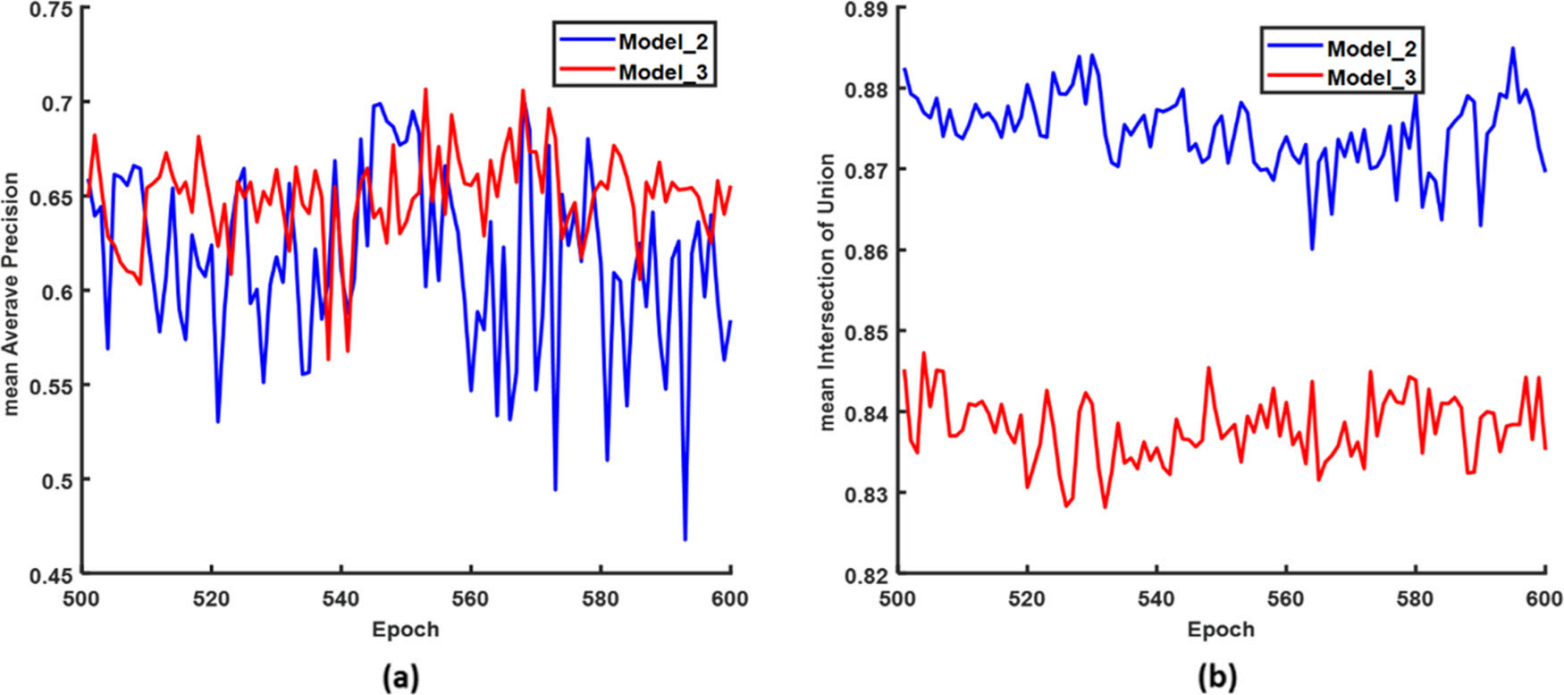
Comparison of Model_2 and Model_3 for epoch 501–600 inferencing on 20 test images. **a** Mean average precision and **b** mean intersection over union.

Inferencing of 20 images by both Model_2 (the model trained by 88 images with generated annotation based on Model_1, shown in Fig. 8) and Model_3 (the model trained by 88 images with manual annotation based on Model_1, shown in Fig. 8) confirmed the accuracy of the generated mask with manual corrections, while the Model_1 was the initial model trained by 137 images with manual annotation. The mAP and mIOU with epochs during 501–600 of Model_2 and Model_3 with a 0.5 IOU threshold are shown in Fig. 3. With different training epochs, the results varied. Overall, the mAP of Model_3 was better than that of Model_2 in these epochs (Fig. 3a), which meant that the detection and classification accuracy of Model_3 was better than that of Model_2 in most epochs. However, it still can be seen that the mAP of Model_2 was better than that of Model_3 in some epochs, such as 505–509, 525, and 545–551. The result indicates that using generated annotation with manual correction could be as accurate as manual annotation. The highest AP of Model_3 was 0.706 in epoch 553, while the highest AP of Model_2 was 0.699 in epoch 546. The difference between these two mAPs was 0.007, indicating that this semi-annotation method was more accurate. Figure 3b indicates that the mean IOU of Model_2 was higher than that of Model_3 in all epochs, which meant that the mask accuracy of Model_2 was higher than that of Model_3 in these 20 testing images. However, the mIOU difference was small (~0.004 in all epochs). In summary, using iterative annotation proved to be a feasible time-saving method^37,38^.

### Blueberry traits analysis

The blueberry traits of 104 samples from four cultivars (‘Emerald’, ‘Farthing’, ‘Meadowlark’, and ‘Star’) were extracted using customized algorithms based on the berry detection and segmentation results. The average values of berry number, maturity, and compactness of five views were used as the traits for the sample. It was determined that all four cultivars were partly mature in mid-April. The ‘Emerald’ had the largest average berry number per branch among the four cultivars, almost twice that of the ‘Star’ cultivar (Table 1). The average berry number per branch of the ‘Farthing’ cultivar was about one berry lower than that of the ‘Emerald’. ‘Meadowlark’ was between ‘Farthing’ and ‘Star’. In mid-April, the average maturity of ‘Meadowlark’ was the highest while ‘Farthing’ was the lowest. The maturity of ‘Emerald’ and ‘Star’ was slightly over 0.5, indicating that over half of the berries were blue. The maturity of ‘Farthing’ was a little less than 0.5. The average compactness data showed that ‘Farthing’ had the most compact blueberry clusters, which suggests that it is the most difficult to harvest for the first harvest of the season and ‘Meadowlark’ is the easiest. These results indicate that ‘Meadowlark’ and ‘Star’ are the least compact and most mature, so they are ready to harvest. However, the average value for all of the cultivars did not represent a single case. It is necessary to do statistical analysis for all of these blueberry traits for all four cultivars.

**Table 1:** Average traits extracted by the imaging method for four cultivars.

Cultivar	Average berry number per bunch	Average maturity	Average compactness
‘Emerald’	13.861	0.528	0.595
‘Farthing’	12.897	0.477	0.647
‘Meadowlark’	11.394	0.651	0.514
‘Star’	8.534	0.574	0.574

The average traits refer to the average traits of 26 samples for each cultivar.

**Fig. 4:**
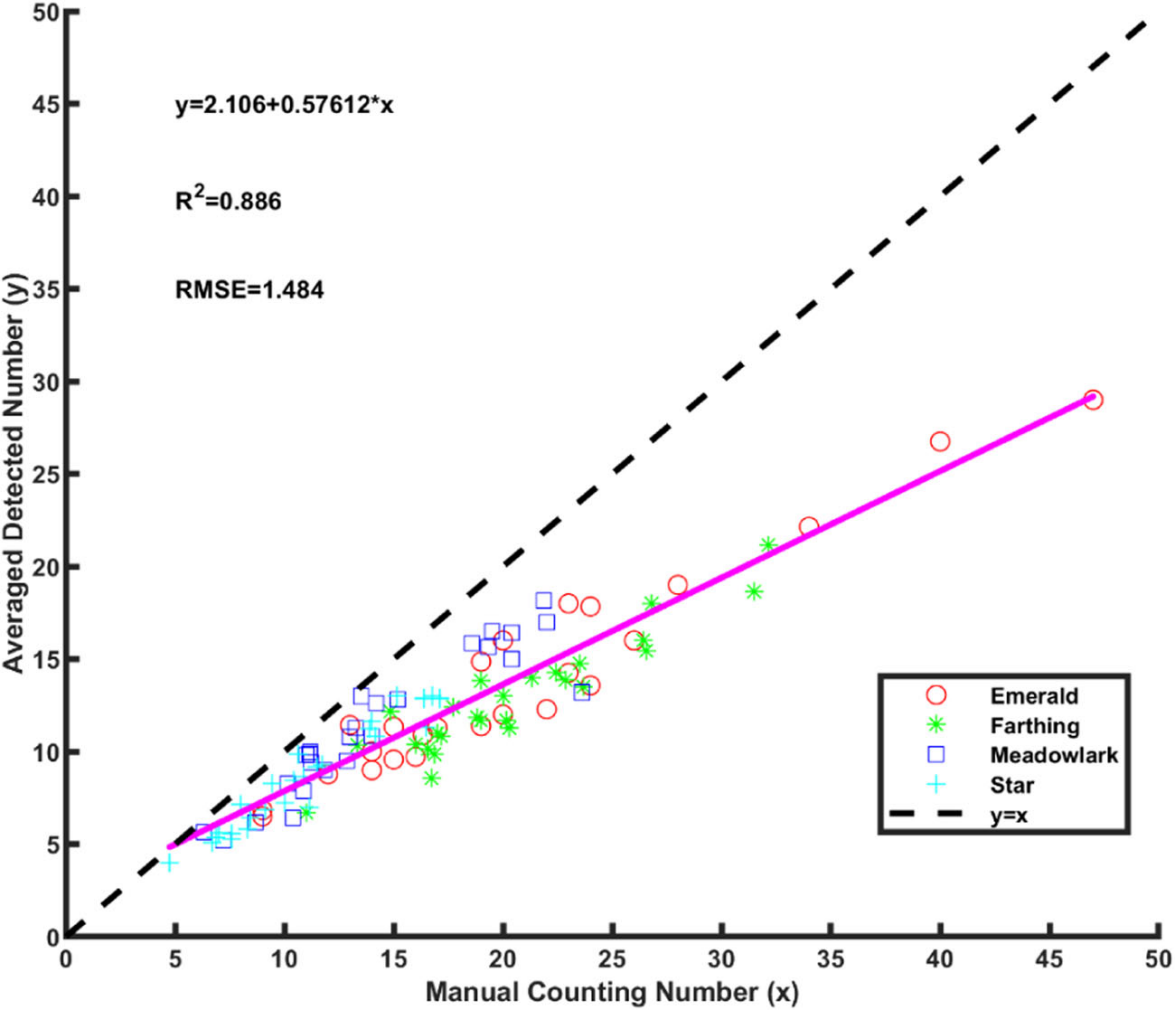
Linear regression of detected number with ground truth for all cultivars. Different markers represent different cultivars.

The number of blueberries within a branch was evaluated with the ground truth, as the number can potentially be used for yield prediction. Figure 4 shows the linear regression for the detected number with the manual counting number for all of the cultivars. Overall, berry number by counting every segmented berries was underestimated compared with the ground truth. This is because not all of the blueberries can be seen in a single 2D image, and the error increased as the number of berries increased because more berries became hidden by fruit in the foreground. A linear model was obtained between the ground truth and the detected number with a coefficient of determination (*R*^2^) of 0.886 and a root mean square error (RMSE) of 1.484. The results of linear regression analysis for the four cultivars compared with the four cultivars combined are presented in Table 2. ‘Star’ had the highest *R*^2^ and the lowest RMSE. ‘Meadowlark’ had the lowest *R*^2^ in comparison with the ground truth. ‘Emerald’ had an *R*^2^ of 0.93 and the highest RMSE, indicating that the linear model for ‘Emerald’ had the largest error. Compared with the linear model for the four cultivars combined, all of the linear models for a single cultivar had a lower RMSE. ‘Emerald’ and ‘Star’ had a higher *R*^2^ compared with the four cultivars combined. The results suggest that the use of single cultivar linear model is a better predicator of blueberry number than the combined cultivar linear model.

**Table 2:** Linear regression for four individual cultivars.

*h*	Linear model	*R*^2^	RMSE
‘Emerald’	*y* = 1.296 + 0.606 × *x*	0.932	0.715
‘Farthing’	*y* = 1.145 + 0.575 × *x*	0.877	0.548
‘Meadowlark’	*y* = 1.124 + 0.719 × *x*	0.859	0.693
‘Star’	*y* = 0.144 + 0.768 × *x*	0.934	0.359

**Fig. 5:**
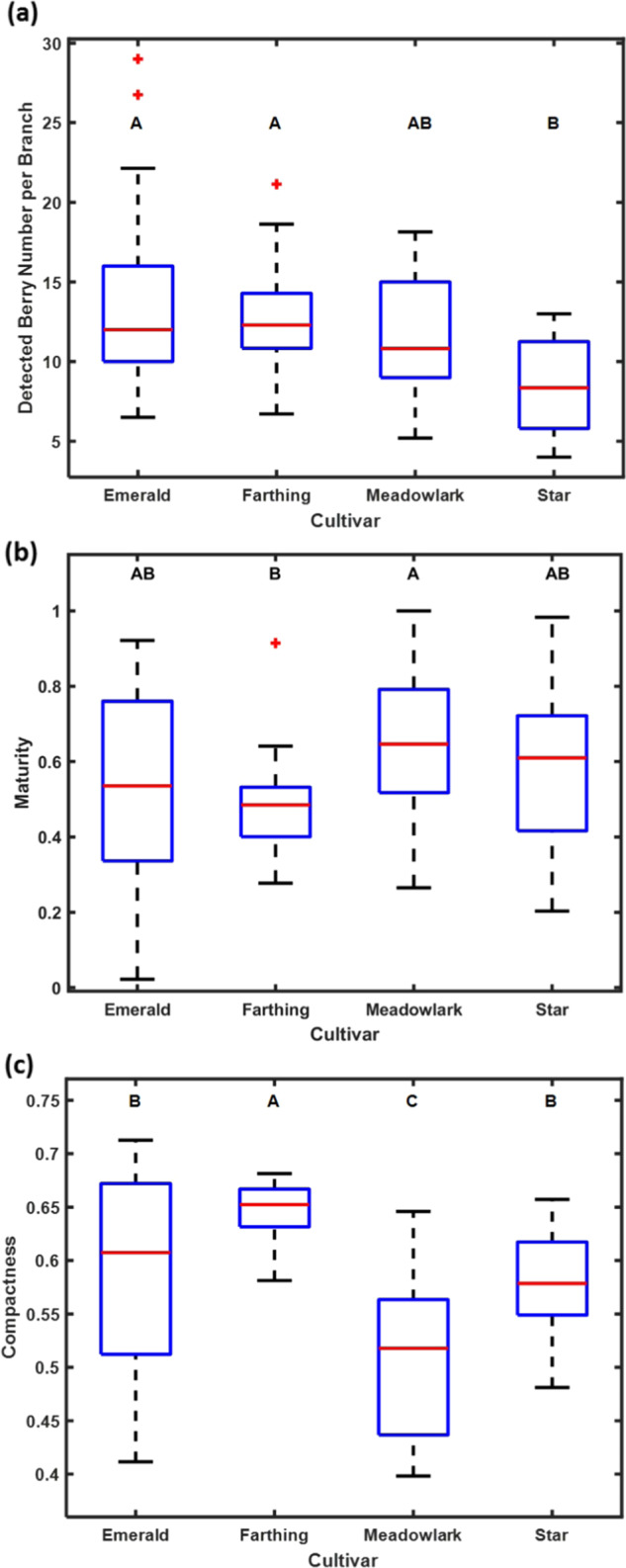
Statistical analysis of the traits with different cultivars. **a** Total blueberry number within each branch. **b** Maturity. **c** Compactness. Two cultivars with the same letter means they are in the same group without significant differences in that trait. The red plus symbol represents the outliers of the dataset.

Statistical analyses revealed trait differences among these four cultivars. The berry number per branch of ‘Star’ was significantly lower than that of ‘Emerald’ and ‘Farthing’, while that of ‘Emerald’, ‘Farthing’, and ‘Meadowlark’ were similar (Fig. 5a). ‘Star’ has a lower berry number per branch than ‘Emerald’ and ‘Farthing’. The analysis for fruit maturity indicated that ‘Meadowlark’ was further along in the fruit maturation than the other three cultivars, as shown by the greater number of blue fruit in a branch (Fig. 5b). For compactness, only ‘Emerald’ and ‘Star’ have no significant difference. ‘Farthing’ has the highest compactness, ‘Star’ and ‘Emerald’ are the second, and ‘Meadowlark’ is the lowest (Fig. 5c). This indicates that ‘Meadowlark’ is the best cultivar among these four for machine harvesting for the first harvest of the season.

## Discussion

### Detection error

**Fig. 6:**
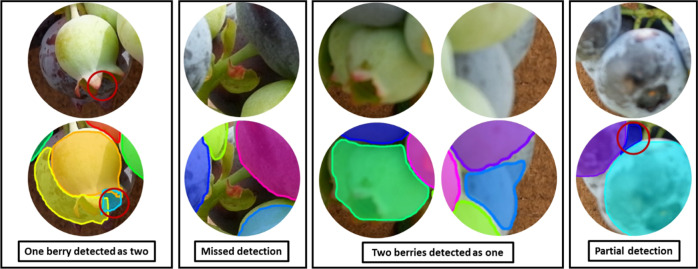
Four types of detection errors. They are one berry detected as two, missed detection, two berries detected as one, and partial detection, respectively.

There are four main types of detection errors: one berry detected as two, missed detection, two berries detected as one, and partial detection, as illustrated in Fig. 6. For one berry detected as two, the example shows a small part of a berry wrongly detected as an individual berry and another part of this berry detected as a separate berry. For missed detection, some berries are occluded by the stem, leaves, and other berries, and these berries are difficult to be detected even by human eyes. When two berries are not separated clearly and one berry is partly covered by the other berry, they are more likely to be detected as one berry. For partial detection, most parts of the berry were covered by two other berries and the visible part was divided into two unconnected parts. Predictably this berry was only detected partially and another visible part of this berry was not detected, possibly because of the small area that was visible to the camera. Because of this kind of error, the mask accuracy was decreased. In other cases, some leaves also were detected as immature berries. The most probable reason for these inaccurate detections is that there are many berries covered by each other, resulting in only small parts of some berries being visible. Although there were some detection errors caused by the inherent limitation of the 2D images, the overall results were promising (Fig. 1).

### Maturity

**Fig. 7:**
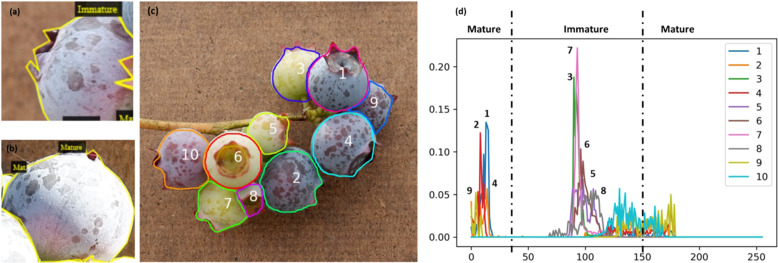
An objective method to assess fruit maturity. **a**, **b** Inconsistency of annotation in maturity. **a**, **b** Appear similar color, but **a** was annotated as immature, **b** was annotated as mature. **c**, **d** Blueberry maturity detection using Hue based on detected berries. **c** shows berries detected by the detection model and **d** shows the maturity detection result using the Hue histogram. The horizontal axis in **d** is the Hue value and the vertical axis in **d** is the percentage of the pixel number of corresponding Hue value. When 0 < Hue value < 30 or 150 < Hue value < 180, then defined as mature.

One important reason for the classification error is inconsistency in maturity annotation. Figure 7 shows an example of these inconsistencies, where berries in (Fig. 7a) and (Fig. 7b) show a similar color, but berry in (Fig. 7a) was labeled as immature, berry in (Fig. 7b) was labeled as mature. Because of the inconsistency in annotation, the trained model may have incorrectly detected the maturity of berries. To make the maturity results become consistent, some objective methods may be needed to determine maturity. This study explored one objective method. The color of each detected berry was extracted to be represented as the berry color, but there were two problems: (1) the images were taken outdoors and the illumination of each image varied; and (2) the detected berry mask might have contained some parts of other berries. To solve these two problems, the Hue value (not considering saturation and illumination) was used instead of RGB color, and the boundary of every mask was decreased by 10 pixels (to remove the background near the boundary). Figure 7c shows the results of this method used to assign maturity rating to ten berries on a branch. The mature berries were No. 1, 2, 4, 9, and the immature berries were No. 3, 5, 6, 7, 8, and 10. Hue value is an objective method of determining berry maturity and is superior to the subjective, visual annotation method.

### Blueberry number and cluster number

The number of clusters on a blueberry branch is a factor of compactness difference when cultivars have the same blueberry number on a branch. A blueberry branch can have several clusters, and each cluster has several blueberries. It is easy to distinguish loose clusters but difficult to distinguish compact clusters. In this way, blueberry compactness of the entire branch is also affected by the number of clusters. The blueberry branch would be more compact if there were fewer clusters when the number of blueberries is the same. However, it is also difficult to detect the cluster numbers on some cultivars. The cluster number was recorded by destructive methods. The average cluster numbers per branch of ‘Emerald’, ‘Farthing’, ‘Meadowlark’, and ‘Star’ were recorded as 3.759, 2.678, 3.611, and 1.801, respectively. Combined with the average compactness in Table 1 and Fig. 5c, it can be inferred that the compactness of ‘Star’ was higher than ‘Meadowlark’, although there was no significant difference in the number of blueberries between them because the average cluster number of ‘Star’ was only half that of ‘Meadowlark’. It also can be inferred that the compactness of ‘Farthing’ is higher than ‘Emerald’ while they have no significant differences in berry number.

### Harvestability traits

There are many factors affecting blueberry mechanical harvestability, i.e., bush architecture, harvest time, and the detachment difference of mature fruit and immature fruit^39,40^. Compactness is one factor contributing to harvestability. The assumption in this paper, ‘less compact is more suitable for mechanical harvesting,’ is based on when other factors are the same and the harvest time is specifically for the first harvest of the season.

Maturity can be explained as harvestable. In this paper, there are two indices used to describe fruit maturity. One is for the individual berry maturity. Another is the maturity ratio (mature berry number/total berry number) for the whole clusters. Individual berry maturity can indicate whether the berry is harvestable or not. The cluster maturity ratio can indicate whether or not this cultivar can start harvesting at a specific time.

## Advantages and limitations

This study presents a deep learning model for individual blueberry detection and segmentation for 2D images of blueberries in situ in the field. Furthermore, blueberry harvestability and yield traits (berry number, maturity, and compactness) were extracted directly. Existing methods cannot extract all of these traits, particularly compactness for blueberries^7,8,35,41^. Although the traits were extracted from 2D images, the analyses were performed with five different views of clusters on a branch. The use of multiple images of clusters for extracting traits of interest eliminated potential outliers from one particular viewing angle. The berry count error was mainly caused by occlusion, an inherent limitation with 2D images. Therefore, it will be necessary to extract traits from 3D images in the future^9,42^. In addition, the methodology described in the present study was unable to determine the contribution of pedicel length on cluster tightness.

## Conclusions

This study investigated a deep learning segmentation model Mask R-CNN for segmenting individual blueberries and extracting harvestability and yield traits for 2D images of blueberries in situ in the field. The classification accuracy (mAP) was better than 0.7 at an 0.5 IOU threshold with a 0.9 segmentation accuracy (mIOU). The blueberry numbers extracted from this model showed that ‘Star’ has a lower berry number, ‘Farthing’ is less mature in the middle of April but is most compact, and ‘Meadowlark’ is the loosest in comparison with the other three cultivars. The trait of blueberry number have potential applications for yield estimation with maturity. The compactness analysis can be incorporated into a breeding scheme in which genotypes are selected for machine harvestability. In future studies, 3D imaging of individual berries will be segmented to obtain more precise quantification of fruit and cluster traits.

## Materials and methods

The study was conducted in two parts. Section ‘Individual blueberry detection and segmentation’ involved the development of detection and segmentation of blueberry fruit for building a model for individual blueberries using Mask R-CNN. Section ‘Blueberry traits extraction and analysis’ included the extraction of blueberry fruit traits based on this model. Two different image datasets were collected. The dataset used in Section ‘Individual blueberry detection and segmentation’ was used for training to obtain a blueberry segmentation model, which was split into training, validation, and testing datasets. The dataset in Section ‘Blueberry traits extraction and analysis’ are the images used to extract blueberry traits based on the segmentation model for the four different cultivars (‘Emerald’, ‘Farthing’, ‘Meadowlark’, and ‘Star’).

### Individual blueberry detection and segmentation

#### Image dataset

**Fig. 8:**
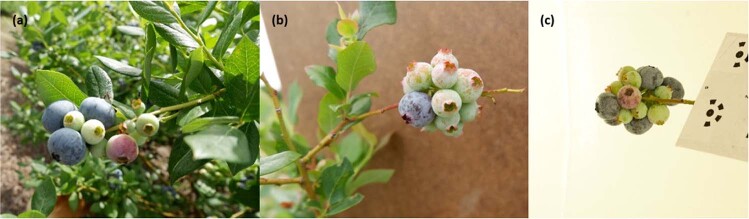
Three conditions of blueberry images for training. **a** With natural background, **b** with artificial background in the field, and **c** with artificial background in the lab. From left to right, blueberries developing on a branch are shown: **a** two or three clusters, **b** unable to determine the number of clusters in situ by visual means, and **c** a branch with several clusters of blueberries.

The Blueberry images were obtained under three different lighting conditions and backgrounds: (1) outdoor lighting with a natural background (Fig. 8a), (2) outdoor lighting with an artificial background (cardboard) (Fig. 7b), and (3) artificial lighting with an artificial background (Fig. 8c). The image dataset produced 724 images: (1) A total of 524 images in the training dataset (482 images with background A, 18 images with background B, and 24 images with background C), (2) 145 images in the validation dataset (126 images with background A, 9 images with background B, and 10 images for background C), and 55 images in the testing dataset (30 images with background A, 20 images for background B and 5 images for background C). The images of conditions (Fig. 8a) and (Fig. 8b) were captured randomly on commercial farms in Alma, Georgia, on April 17th, 2019. Some blueberry bunches were cut and transported to the lab in the University of Georgia. The images of condition (Fig. 8c) were captured in the lab. The total number of images used in the training was 669, which is the sum of the training dataset and the validation dataset.

#### Image annotation

**Fig. 9:**
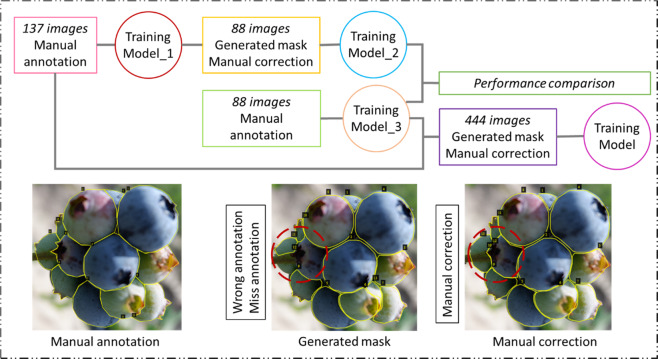
Annotation workflow. 137 and 88 images were manual annotated, the annotation of another 444 images were generated by Model_3 with manual correction. The final Training Model was used to segment blueberries on four cultivars.

Images were annotated with three classes: background, mature berry, and immature berry. Blue-colored berries were labeled as mature berries. Green-, red-, or deep red-colored berries were labeled as immature berries. A VGG Image Annotator was used for image annotation, and polygon was used to outline manually the boundaries of individual blueberries. Initially, 137 images were annotated manually to train a detection model (Model_1) with image augmentation for 300 epochs. A semiautomatic annotation method was used (Fig. 9) to make the annotation more efficient. The annotation of another 88 images was generated by pretrained Model_1. Then the manual correction sought to verify the generated annotation to correct misclassified and misaligned polygons, and to annotate the berries that were not detected by Model_1. To verify that this iterative annotation method was efficient, these 88 images were annotated manually as well. It should be noted that generated annotation with manual correction takes 1 h while fully manual annotation takes 6 h. These 88 images annotated with different methods were trained in the same manner as Model_1 to obtain Model_2 and Model_3, respectively. The performance of Model_2 and Model_3 were compared using 20 images with manual annotation. These 20 images were taken from the images mentioned in Section ‘Blueberry traits extraction and analysis’. Next, the remaining 444 image annotations were generated by Model_3 with manual correction. The annotation used for the testing images was also generated by Model_3 with manual correction.

#### Mask R-CNN based detection and segmentation pipeline

**Fig. 10:**
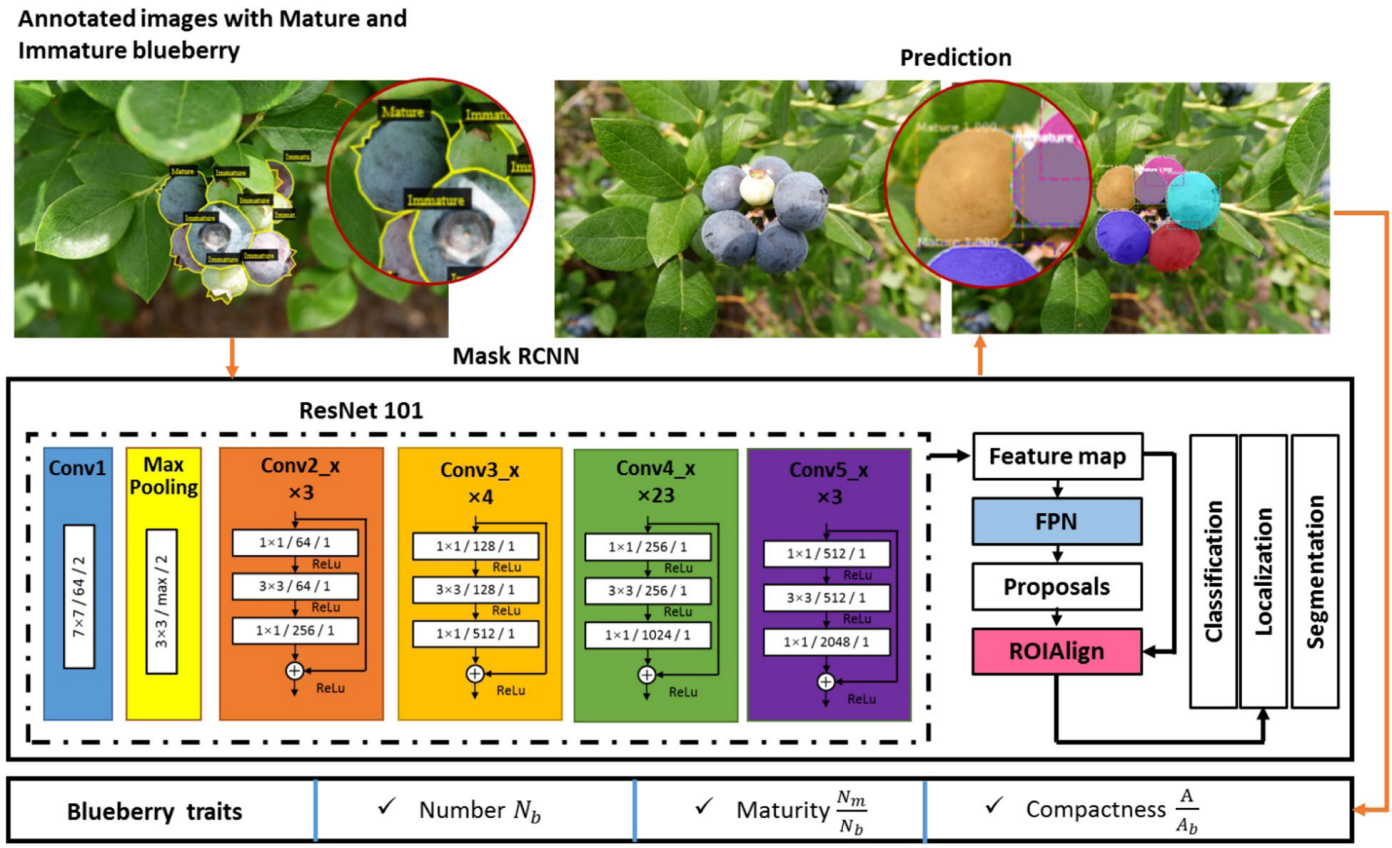
Mask R-CNN based blueberry detection and segmentation pipeline. There are three steps: the annotated images were used to train the Mask R-CNN model, the trained model was used to predict test images, and the traits of fruit number, maturity, and compactness were extracted from the predicted images.

The implementation of Mask R-CNN in this paper uses ResNet101 plus an FPN (feature pyramid networks) backbone to extract feature maps. The RPN (region proposal network) was used to scan the backbone feature map, which reuses the extracted features efficiently and avoided duplicated calculations to get the region of interests (ROIs). The next step used an ROI classifier to classify regions to specific classes and used a bounding box repressor to encapsulate the object. The previous steps involved the implementation of a Faster R-CNN^24^ for object detection. The following step was the additional mask network of Mask R-CNN to obtain the final masks. The Mask R-CNN based blueberry detection and segmentation pipeline is shown in Fig. 10. The image dataset was fed to the Mask R-CNN framework to obtain the detection model. We used the detection model to detect and segment blueberries in the images with mature or immature properties. Next, the blueberry traits (number, maturity, and compactness) were obtained automatically with customized definition and programs.

The training had specific configurations. There were three classes: background, mature berry, and immature berry. The training dataset had 524 images, the validation dataset had 145 images, and 262 and 73 images were used in the training steps per epoch and validation steps per epoch with a batch size of 2. Seven different image augmentation methods were used to increase image conditions: rotate 50% horizontally, rotate 50% vertically, rotate 90°, 180°, and 270°, respectively, multiply with a random value between 0.5 and 1.5, and blur with a Gaussian kernel with a sigma of 5.0. The images were resized to 1024 × 1024. The learning rate was 0.001 with a momentum of 0.9. The loss weight of the RPN class, RPN bounding box, Mask R-CNN class, Mask R-CNN bounding box, and Mask R-CNN mask were the same. These losses were summed to obtain the final loss. The pretrained weights on the MS COCO^43^ were used to initialize the weights of the Mask R-CNN model. The training was done using a Tesla V100 GPU card with 16G memory.

#### Performance evaluation

AP was used to evaluate the classification performance. Precision is the ratio of the true detected objects over the total number of detected objects. A precision of 1 meant that all detections were true blueberries. Recall is the ratio of the true detected objects over the total number of true objects in the image. A recall of 1 meant that all the true blueberries were detected in the image. It should be noted that precision and recall are dependent upon the IOU threshold, which is the area ratio between the intersection and union of the detection and the ground truth. Different IOU thresholds generated different precision and recall. A precision-recall curve was obtained by using the average values of the precision across all recall values^44^. To accurately analyze the detection and classification performance, an AP with different IOU thresholds between 0.5 and 0.95 with 0.05 interval was calculated. Then, the mAP over all the blueberries in the dataset was measured. The detected mask area was used to determine blueberry cluster compactness trait. Thus, the mean IOU was calculated to indicate mask segmentation accuracy.

### Blueberry traits extraction and analysis

#### Blueberry samples

**Fig. 11:**
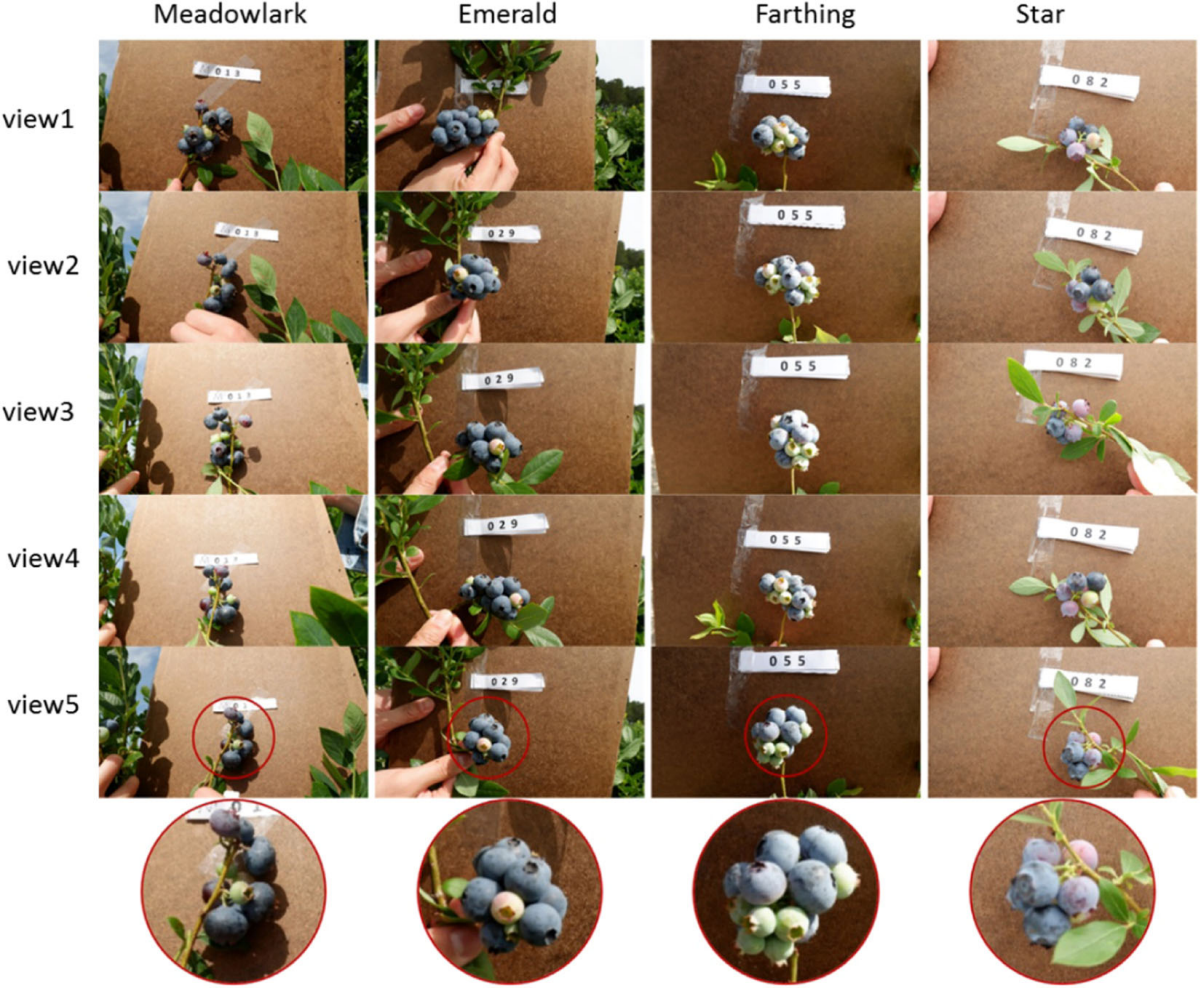
Illustration of blueberry clusters from different views. Four different cultivars (‘Meadowlark’, ‘Emerald’, ‘Farthing’, and ‘Star’) were imaged with five different views (by rotating the fruit cluster 360°).

The blueberry samples for trait analysis were imaged using a DSLR (digital single-lens reflex) camera (X-A10, Fujifilm, Japan) with a resolution of 4896 × 2760 pixels. The images were collected from mature blueberry plants on commercial farms in Alma, Georgia on April 17th, 2019, when the fruit was partly mature and not yet harvested. Four blueberry cultivars (‘Meadowlark’, ‘Emerald’, ‘Farthing’, and ‘Star’) with 26 branches per cultivar and 5 views per branch (covered 360°) were captured, making the total number of 104 samples with 520 images. All of the images were captured with an artificial background (a brown file folder) to avoid disturbance from other blueberry branches. Figure 11 shows one branch sample for each cultivar. The berries on the ‘Meadowlark’ and ‘Star’ varieties are relatively loose, while those on the ‘Emerald’ and ‘Farthing’ are relatively compact. The total number of berries *N*_b_ and the number of clusters *N*_c_ on each branch were recorded.

#### Traits definition and extraction

**Fig. 12:**
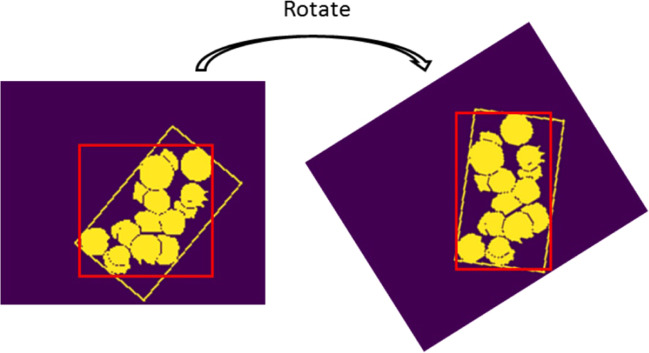
Blueberry with a regular bounding box and minimum bounding box. The yellow circles are the blueberry masks. The yellow rectangle is the minimum bounding box, the red rectangle is the regular bounding box whose sides are always horizontal or vertical.

In this paper, the blueberry traits evaluated are the number of blueberries within a branch (*N*_b_), maturity, and compactness. Based on the Mask R-CNN blueberry detection model, these traits were obtained using the customized definitions and algorithms. *N*_b_ is the number of detected blueberries from the image. Maturity is defined as the ratio of the number of mature blueberries (*N*_m_) over the total detected number (*N*_b_). The compactness is defined as the ratio of the detected blueberry mask area (*A*) over the minimum bounding box area (*A*_b_). The minimum bounding box is the bounding box that has the minimum area to enclose all the detected blueberries. The minimum bounding box remains the same regardless of the orientation of the blueberries in the image, which means the compactness value is consistent using the minimum bounding box (Fig. 12). All the traits are extracted from five different view images for each sample. The average number, maturity, and compactness for the five views of the same sample were calculated as the traits for that sample.

$$maturity\,=\,\frac{{number\;of\;mature\;blueberries}}{{total\;detected\;blueberry\;number}}\,=\,\frac{{N_{\rm{m}}}}{{N_{\rm{b}}}},$$

$$compactness\,=\,\frac{{etected\;blueberry\;mask\;area}}{{minimun\;bounding\;box\;area}}\,=\,\frac{A}{{A_{\rm{b}}}}.$$

#### Traits analysis

The ground truth of blueberry number within a branch (*N*_t_) was recorded manually when collecting data. Linear regression was used to obtain the relationship between the detected number and the ground truth, and the RMSE was calculated. In addition, analysis of variance was conducted (JMP^®^, 14.1.0, SAS Institute Inc., Cary, NC, 1989–2019) to analyze the differences in blueberry number per branch (*N*_b_), maturity, and compactness among cultivars.
